# A Pilot Study to Evaluate the Relationships between Supine Proprioception Assessments and Upright Functional Mobility

**DOI:** 10.3390/brainsci14080768

**Published:** 2024-07-30

**Authors:** Rachel F. Bellisle, Brian T. Peters, Lars Oddsson, Scott J. Wood, Timothy R. Macaulay

**Affiliations:** 1 Harvard-MIT Health Sciences and Technology, Massachusetts Institute of Technology, 77 Massachusetts Ave., Cambridge, MA 02139, USA; 2 KBR, 2400 E NASA Pkwy, Houston, TX 77058, USA; brian.peters@nasa.gov (B.T.P.); timothy.r.macaulay@nasa.gov (T.R.M.); 3 Department of Rehabilitation Medicine, University of Minnesota, 500 SE Harvard St. SE, Minneapolis, MN 55455, USA; loddsson@umn.edu; 4 Recanati School for Community Health Professions, Ben Gurion University of the Negev, David Ben Gurion Blvd. 1, Be’er Sheva 8410501, Israel; 5 RxFunction Inc., 7576 Market Pl. Dr., Eden Prairie, MN 55344, USA; 6 NASA Johnson Space Center, 2101 E NASA Pkwy, Houston, TX 77058, USA; scott.j.wood@nasa.gov

**Keywords:** proprioception, postural control, bedrest, assessment, balance

## Abstract

Long-duration bedrest impairs upright postural and locomotor control, prompting the need for assessment tools to predict the effects of deconditioning on post-bedrest outcome measures. We developed a tilt board mounted vertically with a horizontal air-bearing sled as a potential supine assessment tool for a future bedrest study. The purpose of this pilot study was to examine the association between supine proprioceptive assessments on the tilt board and upright functional mobility. Seventeen healthy participants completed variations of a supine tilt board task and an upright functional mobility task (FMT), which is an established obstacle avoidance course. During the supine tasks, participants lay on the air-bearing sled with axial loading toward the tilt board. Participants tilted the board to capture virtual targets on an overhead monitor during 30 s trials. The tasks included two dynamic tasks (i.e., double-leg stance matching mediolateral tilt targets over ±3° or ±9° ranges) and two static tasks (i.e., single-leg stance maintaining a central target position). The performances during the dynamic tasks were significantly correlated with the FMT time to completion. The dominant-leg static task performance showed a moderate trend with the FMT time to completion. The results indicate that supine proprioceptive assessments may be associated with upright ambulation performance, and thus, support the proposed application in bedrest studies.

## 1. Introduction

Upright balance requires sensory feedback from the vestibular, visual, and somatosensory systems. However, prolonged body unloading leads to deconditioning in proprioceptive and tactile functions, which are critical for postural and locomotor control, resulting in impaired upright functional performance upon return to upright conditions [1]. The effects of body unloading have been studied after exposure to the microgravity environment in spaceflight, and long-duration bedrest is often used as an analog for the body unloading that occurs during spaceflight. Exposure to these environments with body unloading leads to impaired postural and locomotor control [2,3,4], including changes in postural sway [5], lower-limb kinematics [6,7], and functional task performance [3,4,8,9]. Participants tested in an upright ambulation task after long-duration spaceflight or seventy days of head-down bedrest both showed similar significant decreases in performance [8,9], suggesting that deconditioned proprioceptive and tactile functions contribute to postflight impairments in postural and locomotor control [1] that are independent of the vestibular effects of gravity transitions. Impairments from long-duration spaceflight (approximately 6 months) may last for up to 15 days before returning to approximately baseline performance levels according to a study that tested an upright locomotor task postflight [4]. A long-duration (60 to 70 days) head-down bedrest study using the same task similarly showed recovery to near-baseline levels at 12 days post-bedrest [10]. This required re-adaptation time can introduce fall risks after bedrest or upon return from spaceflight (e.g., during emergency egress).

The deconditioning in both environments (bedrest and spaceflight) prompts the need for assessment tools to understand the risks to post-bedrest or postflight upright functional performance. Proprioceptive assessment capabilities could be used for tracking changes over time, prescribing and adjusting countermeasures, and predicting ambulation risks for patients transitioning from long-duration bedrest or astronauts returning from a microgravity environment.

Altered proprioceptive and tactile functions are known to contribute to balance impairments. For example, removing proprioceptors in animal models severely compromises their gait [11]. In patients, various training programs are used to target improvements in proprioceptive and tactile functions. A recent review showed that various proprioception training programs have improved somatosensory function measures in patients with ankle injuries, stroke, and other neurological diseases [12]. In astronauts, postflight hypersensitivity on the foot sole skin has been associated with greater reductions in postural equilibrium scores [13], which is an example of a somatosensory assessment tool. Therefore, proprioceptive and tactile functions may be critical targets for inflight and in-bed sensorimotor assessments.

A tilt board device was proposed by the NASA Johnson Space Center as an in-bed proprioceptive assessment for long-duration bedrest [1]. The tilt board is used with a Gravity Bed to allow for supine in-bed measurements and to minimize vestibular contributions. The Gravity Bed allows participants to lie supine on an air-bearing sled that moves freely on a horizontal surface [14,15,16] while loaded axially with their feet on a vertically mounted tilt board. The Gravity Bed was originally developed for patients with limited upright capabilities or a high risk of re-injury (e.g., spinal cord injury or hip fracture recovery) as an alternative to gait training with partial body weight support, which, due to the required harness, minimizes postural adjustments that are important for gait training [14]. In the context of the current tilt board configuration, the Gravity Bed was used to provide a balance challenge in the supine position, to minimize risk for future bedrest patients, and to reduce upright disruptions to bedrest studies with healthy participants. Previous studies showed that 4 weeks of supine balance training in the Gravity Bed led to increased upright balance performance and increased somatosensory utilization [14]. Gravity Bed balance tasks in acute scenarios also indicated that supine eyes-closed unipedal stance tasks produced greater reliance on somatosensory information than vestibular information [16], supporting the use of the Gravity Bed in the training of proprioception function.

The tilt board is coupled with visual feedback to provide a proactive balance task, where the participant controls the tilt board. The passive tilt board is instrumented to record the tilt angle and enable visual feedback indicating the tilt board’s angle via a cursor on a computer display. This visual feedback guides participants to make proactive tilts that control the tilt of the board to move the feedback cursor toward virtual targets. Similar use of visual feedback was shown to be effective in upright balance training and assessments [17,18,19,20]. In the supine position, visual feedback can be used to direct external goals for motor and balance control, and support the connection between internal proprioception feedback and motor output [1].

The goal of this work was to address a research gap: to develop an assessment tool to characterize and monitor the effects of long-duration bedrest on postural and locomotor control. The tilt board is proposed as a candidate technology to address this research gap and may provide a proprioceptive assessment capability to monitor proprioception ability over time. Validation is required through participant studies, and the anticipated trajectory of investigation for the tilt board technology includes pilot testing in acute unloading settings, followed by longer participant studies to assess use during chronic unloading and body deconditioning, which will be completed in an upcoming head-down bedrest study [21].

Since effectiveness as an assessment tool is critically tied to post-bedrest ambulation, a pilot study, which is presented in this paper, was necessary to determine which supine tilt board task conditions best correlated with upright functional mobility. We investigated the use of the tilt board device as a proprioceptive assessment tool during an acute supine body position on the Gravity Bed. The tilt board did not measure proprioception directly, but it measured performance on a task that required proprioceptive function. This study aimed to answer the following research question: what is the association between supine proprioceptive assessment scores on the tilt board and upright functional task performance? We hypothesized that participants who performed better on the tilt board would also perform better during upright functional performance. This would support the use of the tilt board as an in-bed assessment tool.

## 2. Materials and Methods

### 2.1. Participants

This study was approved by the Institutional Review Board at NASA Johnson Space Center. Seventeen healthy participants provided informed consent and completed the study within a single session. The sample size was based on data from a previous study with 14 participants that found Bonferroni-corrected differences between eyes-open and eyes-closed conditions using an earlier version of the Gravity Bed [16].

The participant characteristics are included in Table 1. Seven participants had previous tilt board experience (≥3 sessions) from pilot testing used to determine the assessment parameters, including the axial loading level, virtual target locations, and foot positions. Sixteen participants reported right foot dominance, with the dominant foot defined as the foot used to “kick a ball or write their name in the sand”.

### 2.2. Assessment Overview

The participants completed four proprioception assessments, with two trials completed for each activity. One upright activity, the functional mobility task (FMT), was used to represent upright functional performance, and three supine activities were completed on the Gravity Bed, including two variations of a double-leg dynamic task (DY) on the tilt board and a single-leg static task (ST), which was repeated for each leg, on the tilt board. All supine tasks were completed without shoes or socks. The participants wore sneakers or other comfortable athletic shoes during the upright tasks only. The use of shoes is consistent with previous studies and maintains ecological validity for upright operations.

### 2.3. Assessment Activities

**Figure 1:**
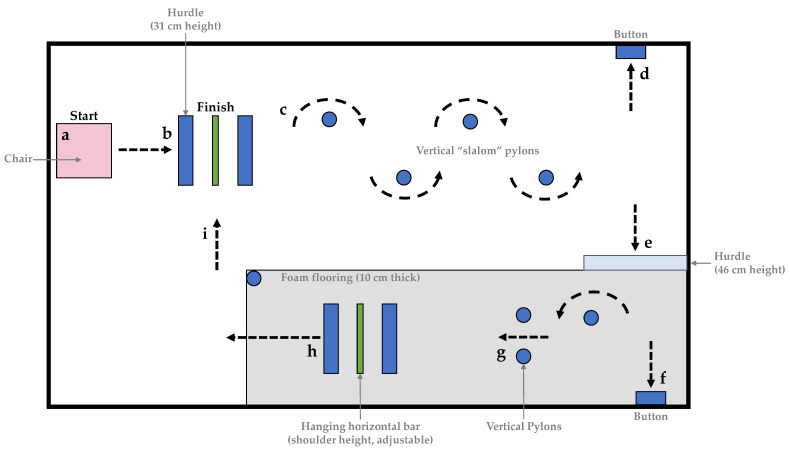
Functional mobility task (FMT). The participant (**a**) starts seated in a chair, and when prompted to start, (**b**) steps over 31 cm tall obstacles and under a horizontal bar hanging from the ceiling at shoulder height. (**c**) They weave through 4 vertical pylons and (**d**) press a button mounted on the wall. (**e**) They step over a 46 cm tall obstacle onto a surface of 10 cm thick medium-density foam and (**f**) press another button mounted on the wall. (**g**) They pass through a gate formed by two vertical pylons and (**h**) step over another set of 31 cm tall obstacles and under a hanging horizontal bar. (**i**) After stepping off of the foam floor, they turn right and pass between two hurdles to finish the task. The diagram is not to scale. Figure modified from [22].

The FMT is a standard measure designed to characterize locomotor function and consists of an obstacle avoidance course, which is described in prior literature (Figure 1) [22]. The course is set up in a 6.0 m by 4.0 m area and requires participants to duck under, step over, weave through, and turn around obstacles. Half of the course includes walking on a 10 cm thick medium-density foam to add a proprioceptive challenge. The obstacles in the FMT present several types of sensorimotor challenges, including dynamic single-leg balance while stepping over hurdles, full body segmental coordination while walking around and ducking under obstacles, multiple changes in heading, and proprioception disruption with foam flooring [4,9]. Participants were instructed to complete the activity “by walking as quickly and safely as possible without touching any obstacles”. The FMT time to completion was recorded.

**Figure 2:**
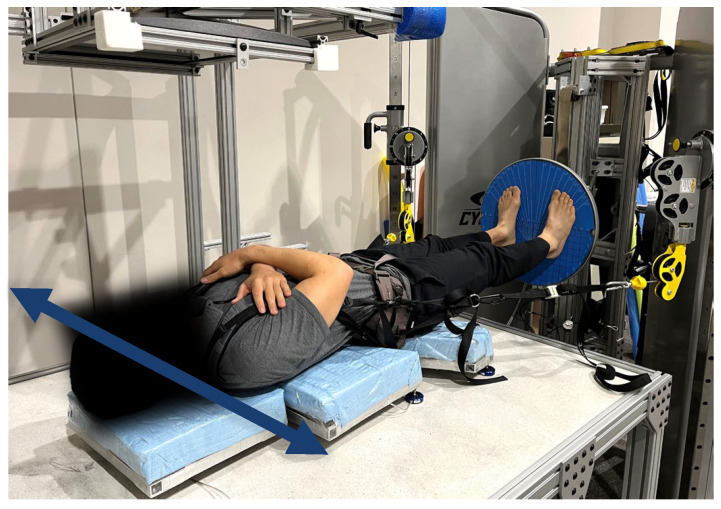
The Gravity Bed. The Gravity Bed is used to allow for a proprioceptive challenge in a supine body orientation [14,15,16] and can be used in combination with the tilt board. The participant lays on a two-piece sled on air bearings, which allows for mediolateral movements (indicated with an arrow) with minimal friction. An axial body load toward the feet is provided by an adjustable weight and pulley system, which is attached to lateral carabiners on a harness worn by the participant.

**Figure 3:**
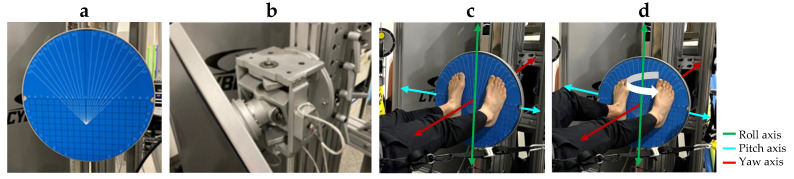
The tilt board. The tilt board is proposed as a supine proprioceptive assessment tool. It includes (**a**) a balance board surface with positioning reference markings and (**b**) a passive balance board gimbal mechanism that allows for up to 20 degrees of tilt in the anterior–posterior (i.e., pitch) and mediolateral (i.e., roll) directions simultaneously, with instrumentation to record tilt angles to use in the visual feedback display. (**c**) Axes of rotation are displayed with the tilt board at a 0° position. (**d**) As an example of tilt board movement, the white arrow indicates a roll movement toward the right foot.

The Gravity Bed was used to allow for balance challenges in a supine body orientation [14,15,16]. The Gravity Bed consists of a horizontal surface and a padded two-piece sled that moves freely on air bearings. The participant is secured in a harness while lying supine on the sled on the Gravity Bed surface (Figure 2). The harness is attached to an adjustable weight stack pulley system to provide a load toward the feet along the longitudinal axis of the participant’s body. In this configuration, the Gravity Bed allows for frontal plane (i.e., mediolateral) motion with minimal friction during a supine stance on the tilt board (Figure 3), which was mounted perpendicular to the Gravity Bed surface.

During the tilt board tasks, tilt board angle feedback was provided as a cross-hair cursor on a monitor mounted above the subject. Green targets appeared on the screen, and the participant was instructed to move the cursor and capture targets by tilting the tilt board. During the trial, the tilt board pitch and roll were recorded with an instrumented gimbal, with a built-in 7-sample moving average. Before the start of the tilt board data collection, participants were allowed a familiarization session, including one full activity profile with 14 targets ranging from −15° to +15°.

In the DY tilt board variations, the goal was to capture multiple targets with both feet placed together on the tilt board and arms folded over the chest. The Gravity Bed harness was loaded with 60% of the participant’s self-reported body weight. The participant was instructed to capture as many green targets as possible in 30 s, after initiating the trial by capturing the first target. This was accomplished by holding the cursor on the first target within a ±6-pixel (±0.27°) accuracy window for a 0.5 s dwell time. To capture subsequent targets and move on to the next, the participant needed to hold the cursor within the same accuracy window for a 0.75 s dwell time.

**Figure 4:**
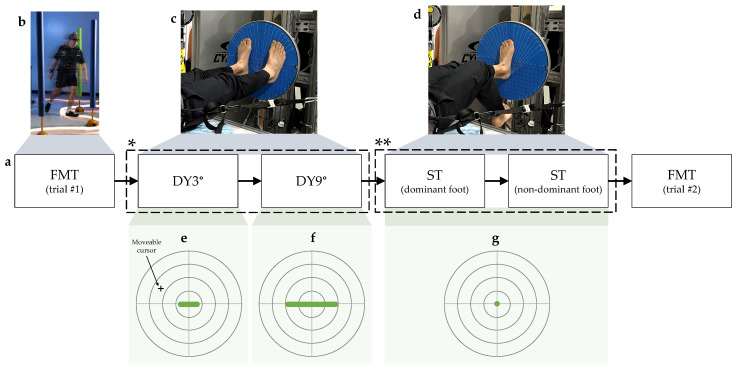
An overview of the assessment activities and experiment design. (**a**) The data collection session started and ended with an FMT trial, and the tilt board activities were presented in a sequence that became progressively more challenging. * The DY trials were alternated between the DY3° and DY9° until two trials were completed for each. ** The ST trials were alternated between the left and right foot until two trials were completed for each. (**b**–**d**) show photos of each assessment activity, including (**b**) the FMT, (**c**) double-leg stance for the two DY activities, and (**d**) single-leg stance for the ST. (**e**–**g**) show examples of tilt board performance feedback for each activity, as displayed on a computer monitor. A cursor was controlled by the tilt position of the board. The objective was to move the cursor toward a green target highlighted on the screen. The target locations varied across the 3 tilt board activity types used in this study, namely, the (**e**) DY3°, (**f**) DY9°, and (**g**) ST. Green markings indicate the possible locations of targets in each activity, including a range of possible target locations for the DY3° and DY9°, and one center target for the ST.

Although it was beyond the scope of this investigation to understand the effects of magnitude and direction on the supine postural assessment task, we used two dynamic activity variations (DY3° and DY9°) with different tilt amplitudes to explore how their associations with the upright performance differed. Both activity variations were comprised of seven possible targets in the participant’s frontal plane, which prompted mediolateral tilts of the board. In the first variation (DY3°), the possible targets varied from −3° to +3° at 1° intervals in the mediolateral direction (Figure 4e). In the second variation (DY9°), the targets varied from −9° to +9° at 3° intervals in the mediolateral direction (Figure 4f). Each of the six possible non-zero targets was presented in a random order, followed by a return to the center target before any target locations were repeated within a 30 s trial. For each activity, the task profile (target order and placement) was altered for the second trial to minimize learning effects.

In the ST tilt board variation, the goal was to maintain a static center position. The participant placed one foot on the tilt board for the single-leg stance, and the Gravity Bed harness was loaded with 30% of the participant’s self-reported body weight. A single green target appeared on a screen in front of the participant (Figure 4g), and the participant was instructed to keep the cursor on the green target in the center of the screen for the entire 30 s trial. The trial started after holding the cursor on the first target within a ±6-pixel (±0.27°) accuracy window for a 0.5 s dwell time. Several participants were unable to start the ST due to nearly immediate falls (defined as a ≥20° tilt board position) or an inability to complete the starting dwell time. These participants were allowed several minutes to attempt to start the task before the trial was skipped. They were offered a second chance to start the task in the second trial.

### 2.4. Experiment Design

Two repetitions of all activities and conditions were completed in a single testing session, starting with one FMT trial. Next, the first DY3° and DY9° trials were completed, followed by the second trial of each, respectively. The ST trials were completed next, alternating between the left and right feet until two trials were completed for each. For single-leg tasks, the starting foot (left or right) was randomized to minimize the effects of fatigue. Otherwise, the order of the tilt board tasks was consistent across participants, and was in a sequence that became progressively more challenging, from the DY3°, the least challenging, to the ST, the most challenging. The second FMT trial was completed last to allow for a comparison between the trials to observe any fatigue effects (Figure 4).

### 2.5. Data Processing and Analysis

Time series data for the DY were used to identify how many targets were acquired during the 30 s trial based on the accuracy window and dwell time. The time series data for the ST were used to determine the time on target as a percentage of the total trial time. Any tilt measurements ≥20° indicated that the tilt board “bottomed out”, and the participant lost control of their balance. Therefore, a fall was recorded if the pitch or roll measurements exceeded this 20° limit, and any trials exceeding 19° tilt were manually reviewed. If a fall occurred before 30 s, the trial was terminated, and the time-on-target score was reported as a percentage of the total 30 s.

### 2.6. Statistical Analysis

Before the statistical analysis, performance measures were averaged across the two trials, resulting in one score per participant for each activity. The data were not normally distributed for one of the tilt board activities, as determined with a Shapiro–Wilk normality test, and thus, non-parametric methods were used. Associations between the performance in each activity were determined with Spearman’s rank-order correlations. Visual inspection of the scatterplots for each comparison showed that relationships were generally monotonic. Mann–Whitney U-tests were used to compare the groups of participants with and without prior tilt board experience. Paired samples *t*-tests were used to compare the FMT time to completion across the two trials. The FMT data were normally distributed, as assessed by Shapiro–Wilk’s test, and there were no outliers identified, as assessed by inspection of the boxplots.

## 3. Results

### 3.1. Data Availability and Falls

All participants completed all trials of the FMT, DY3°, and DY9° without any observed falls. The DY9° was not performed by two participants due to a protocol change, and thus, they were omitted from the analysis of the DY9° activity. The fourth task, the ST, was the most difficult. Four participants fell (i.e., bottomed out the tilt board) during one of their ST trials. Additionally, twelve ST trials across five participants were not completed due to an inability to start the task. If one trial was completed, that trial score was used to represent the participant’s average score for the activity; otherwise, the activity was assigned a score of 0%.

### 3.2. FMT Time to Completion

The FMT time to completion was higher during the first trial (21.1 ± 3.6 s) at the start of the data collection session than the second trial (20.0 ± 3.5 s) at the end of the session, with a statistically significant decrease of 1.1 s (95% CI 0.56 to 1.55), t(17) = 4.50, *p* < 0.0005, and d = 0.30. The data provided are given as the mean ± standard deviation. The decrease in the completion time indicates that learning effects likely had a larger effect than fatigue. Given that these effects were small, the FMT time to completion was averaged across trials for the correlation analysis.

### 3.3. Effect of Prior Tilt Board Experience

Seven subjects in this study were a part of preliminary testing to develop the parameters used in this test (e.g., axial loading levels and target positions). Thus, these subjects had previous experience with the Gravity Bed and the tilt board. The results showed no significant differences between the median scores of the experienced versus inexperienced tilt board users in any tilt board activity, namely, the DY3° (*p* = 0.30), DY9° (*p* = 0.057), ST on the dominant leg (*p* = 0.42), and ST on the non-dominant leg (*p* = 0.47). Therefore, all participants were combined for analysis. For completeness, experienced and inexperienced participants are differentiated in the provided figures.

### 3.4. Correlations between FMT and Tilt Board Activities

**Figure 5:**
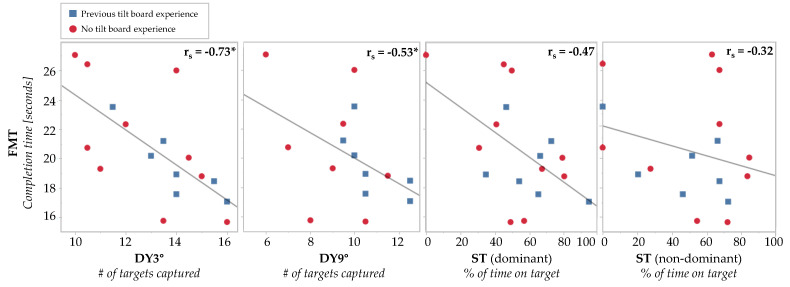
Spearman’s rank-order correlations comparing the relationship between the upright (i.e., the FMT) and supine assessments, namely, (**left** to **right**) the DY3°, DY9°, ST (dominant), and ST (non-dominant). The data show the average performance (across two trials) for each participant. Each plot shows a least squares reference line. The Spearman correlation coefficient is in the upper right corner of each plot, indicating the strength of the correlation. The DY9° was not performed by two participants. * = *p* < 0.05.

When assessing the primary objective of the study, Spearman’s correlations showed statistically significant correlations between the scores for the FMT and each dynamic tilt board activity (Figure 5), namely, the DY3° (r_s_(17) = −0.73, *p* < 0.001) and DY9° (r_s_(15) = −0.53, *p* < 0.05). Spearman correlations between the FMT and ST were not significantly correlated for the ST on the dominant foot (r_s_(17) = −0.47, *p* = 0.058) or the ST on the non-dominant foot (r_s_(17) = −0.32, *p* = 0.20).

### 3.5. Correlations between Each Tilt Board Activity

**Figure 6:**
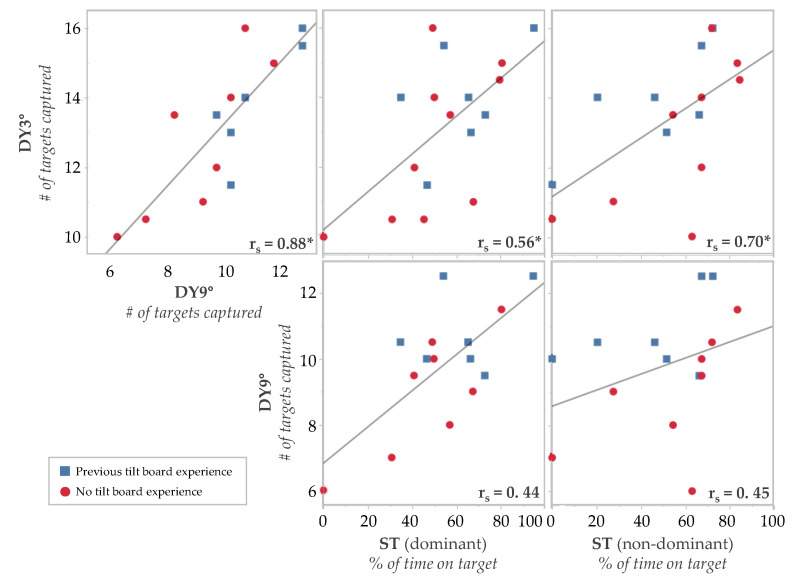
Spearman’s rank-order correlations comparing the relationship between the static and dynamic supine tilt board assessments. The data show the average performance (across two trials) for each participant. Each plot shows a least squares reference line. The Spearman correlation coefficient is in the lower right corner of each plot, indicating the strength of the correlation. The DY9° was not performed by two participants. * = *p* < 0.05.

The static and dynamic tilt board performances were not always correlated with each other (Figure 6). Spearman’s correlations showed that the DY3° had a statistically significant correlation with the DY9° (r_s_(15) = 0.88, *p* < 0.001). There were also significant correlations between the DY3° and the ST on the dominant foot (r_s_(17) = 0.56, *p* < 0.05) and non-dominant foot (r_s_(17) = 0.70, *p* < 0.005). However, the ST was not significantly correlated with the DY9° for the ST on the dominant foot (r_s_(15) = 0.44, *p* = 0.10) or the ST on the non-dominant foot (r_s_(15) = 0.45, *p* = 0.090).

## 4. Discussion

The results indicate that supine proprioceptive assessments on the tilt board may predict upright performance in an operationally relevant dynamic upright task. Dynamic tilt board tasks were significantly correlated with the FMT, which is promising since the FMT is a standard post-bedrest measure designed to characterize locomotor function [4,22]. The DY3° had the strongest correlation to the FMT, suggesting that this configuration or similar double-leg dynamic tilt board variations may be the most appropriate for assessment procedures in the future. This result suggests that the DY3° may have the greatest task-specific relevance to movements during the FMT since it requires active and dynamic bipedal coordination, similar to previous studies, which showed that some non-walking tasks benefit from locomotor training [23].

In addition to the FMT, the DY3° performance was also significantly correlated with all the other tested tilt board activities. These results may indicate that the DY3° has increased robustness compared with other tilt board activity variations, or perhaps it is a more appropriate difficulty level for users with minimal experience on the tilt board. Alternatively, comparisons between different variations of the tilt board task indicate that the dynamic and static assessments may provide different information about sensorimotor performance. For example, the DY9° is not significantly correlated with the ST activity (on both the dominant and non-dominant feet). Different test variations may expose the effects of different balance mechanisms, and a combination of tilt board task types could also be appropriate for an assessment protocol.

While static tilt board tasks were not significantly correlated with upright performance, the correlation between the FMT and ST on the dominant foot was borderline significant (*p* = 0.058), suggesting a larger sample may be required to detect such a relationship. A relationship between the FMT performance and the ST on the dominant foot may indicate that the dominant foot would be more representative of upright functional performance compared with the non-dominant foot. This result differs from the initial hypotheses, which suggested that the non-dominant leg would show higher performance in balance-related tasks due to its typical role in stance and postural support during kicking with the dominant foot. While the ST was defined as “static”, perhaps the movement required more dynamic control, and thus, favored the dominant foot. However, a recent literature review also indicates that balance performance is not significantly different between dominant and non-dominant legs [24].

While this study was not designed to investigate learning effects, a simple statistical test was performed to compare experienced and inexperienced participant groups. Participants with prior experience on the tilt board did not perform significantly better than inexperienced participants, suggesting that the limited previous experience in this study may not provide sufficient training to reveal learning effects in the statistical analysis. Additionally, all participants were provided with familiarization and practice capturing targets using a randomized sequence, which may have provided sufficient learning effects to the inexperienced participants. However, experienced and inexperienced participants were differentiated in the provided plots for transparency, and some evidence of bias in experienced participants is visible in the plots for the DY9°. Despite the observed differences between the experienced and inexperienced participants in the DY9°, the underlying relationship between the tasks was still maintained and similar between the groups, although the experienced participants appeared to show less variability than the inexperienced participants. Limited and irregular use of the tilt board may be representative of a population using the tilt board exclusively as an assessment tool in a bedrest study or operationally inflight. However, if the tilt board has multiple uses as a regular countermeasure and assessment tool, then learning effects could become more prevalent. The effect of previous tilt board experience should be further assessed in future studies.

Possible fatigue effects across the 2 h data collection session should also be acknowledged as a limitation of this study. Fatigue was mitigated with the tilt board activity order (with the most challenging tilt board activities completed last), and for the most challenging task, the single-leg ST, the starting leg was randomized and legs were alternated across trials. Participants were also provided with short rest periods between trials, with the rest duration determined by participant preference. Additionally, the FMT was completed at the beginning and end of the session. The comparison between the two FMT trials allowed for a quantification of fatigue and did not indicate any evidence of fatigue. Participant motivation and effort provide another limitation to consider, which persists across many studies using similar task performance metrics. We assumed that all participants provided their best effort, and the instructions provided in the test administration were consistent across participants.

In this study, all participants were healthy individuals, with no deconditioning at the time of participation beyond possible acute somatosensory effects in the supine orientation [25]. While healthy individuals were appropriate and necessary for pilot testing, future work in the bedrest study will test the effects and feasibility of these activities in deconditioned participants, which will be more representative of future spaceflight or bedrest applications. While short-to-medium-duration head-down bedrest (e.g., 5 to 30 days) can produce balance decrements, these changes are often only observed using the most sensitive assessments, including functional single-leg postures [26,27], electromyographic responses [28], center of pressure derivatives [29,30], or manipulations of all sensory inputs [31,32]. In addition, performance recovers relatively quickly (approximately 3 days). Therefore, longer head-down bedrest durations (e.g., 60 to 70 days) are required for observing balance decreases and for the development of in-bed sensorimotor assessments [8,9,33], and would be most useful for a future tilt board study.

In addition to the relevance to bedrest studies for spaceflight-related research, these findings may have implications for clinical practice. The Gravity Bed was originally invented to support functional rehabilitation in patients who might experience difficult, risky, or less-effective training with full weight bearing or a partial body weight support harness, which is an apparatus that limits some mediolateral postural adjustments that are important for balance and gait training [14,15]. With applicability to upright functional performance, the Gravity Bed and tilt board may be useful in determining the patient’s readiness to progress from supine or partial body weight training to upright functional exercises. Additionally, the tilt board assessment could be used with patients during bedrest to determine when additional rehabilitation or physical therapies are needed to facilitate their future transition to upright activities. In the consideration of risks and benefits when prescribing bedrest [34], supine assessments may also be a useful research tool to measure and monitor the consequences of bedrest over time.

## 5. Conclusions

This work helped to guide the selection of appropriate sensorimotor assessment methods and tilt board configurations for an upcoming bedrest study. The results indicate that performance on the tilt board assessment may be associated with upright functional mobility. A double-leg dynamic tilt board activity, such as the DY3° activity, may be most associated with upright performance. However, future work should continue to explore multiple configurations of the tilt board activity and learning effects. While this pilot study explored acute proprioception assessments with the tilt board, an upcoming study will use bedrest as a spaceflight deconditioning analog to evaluate the tilt board as a proprioceptive assessment system to track proprioceptive function and predict post-bedrest upright performance. This work also supports applications in clinical rehabilitation for the tilt board, coupled with the Gravity Bed, to predict the sensorimotor function and ambulation risks of patients.

## Figures and Tables

**Table 1:** Study participants (*n* = 17).

P#	Sex	Age (Years)	Height (cm)	Weight (kg)	Dominant Foot	Prior Experience
1	F	25	167.6	52.2	R	N
2	F	21	165.1	54.4	R	N
3	M	28	172.7	72.6	R	Y
4	M	24	180.3	79.4	R	N
5	M	22	170.2	72.6	R	N
6	M	22	182.9	88.4	R	N
7	M	25	165.1	90.7	R	N
8	F	24	149.9	52.2	R	N
9	F	22	167.6	56.7	R	N
10	F	27	172.7	59.0	R	Y
11	F	25	157.5	59.0	L	N
12	F	32	167.6	71.7	R	N
13	F	26	152.4	45.4	R	Y
14	M	27	167.6	65.8	R	Y
15	F	30	157.5	56.7	R	Y
16	M	56	167.6	65.3	R	Y
17	M	39	177.8	72.6	R	Y
**Mean**		**27.9**	**167.2**	**65.6**		
**St Dev**		**8.5**	**9.1**	**12.9**		

## Data Availability

The data that support the findings of this study are available upon reasonable request.

## Abbreviations

The following abbreviations are used in this manuscript:

DY	Double-leg dynamic task on the tilt board
DY3°	Double-leg dynamic task on the tilt board with targets ranging from −3° to +3°
DY9°	Double-leg dynamic task on the tilt board with targets ranging from −9° to +9°
FMT	Functional mobility task
NASA	National Aeronautics and Space Administration
ST	Single-leg static task on the tilt board
